# Cord Blood Cardiovascular Biomarkers in Left-Sided Congenital Heart Disease

**DOI:** 10.3390/jcm11237119

**Published:** 2022-11-30

**Authors:** Iris Soveral, Laura Guirado, Maria C. Escobar-Diaz, María José Alcaide, Josep Maria Martínez, Víctor Rodríguez-Sureda, Bart Bijnens, Eugenia Antolin, Elisa Llurba, Jose L. Bartha, Olga Gómez, Fàtima Crispi

**Affiliations:** 1 BCNatal, Fetal Medicine Research Center (Hospital Clínic and Hospital Sant Joan de Déu), 08028 Barcelona, Spain; 2 Obstetrics Department, Hospital General de Hospitalet, 08906 Barcelona, Spain; 3 Pediatric Cardiology Department, Sant Joan de Déu Hospital, Esplugues de Llobregat, 08950 Barcelona, Spain; 4 Cardiovascular Research Group, Sant Joan de Deu Research Institute, Esplugues de Llobregat, 08028 Barcelona, Spain; 5 Laboratory Medicine Department, Hospital Universitario La Paz, 28046 Madrid, Spain; 6 Research Institute IdiPAZ, 28029 Madrid, Spain; 7 Facultat de Medicina i Ciencies de la Salut, Universitat de Barcelona, 08007 Barcelona, Spain; 8 Institut d’Investigacions Biomèdiques August Pi i Sunyer (IDIBAPS), 08036 Barcelona, Spain; 9 Centre for Biomedical Research on Rare Diseases (CIBER-ER), 28029 Madrid, Spain; 10 Catalan Institution for Research and Advanced Studies ICREA, 08010 Barcelona, Spain; 11 Obstetrics and Gynecology Department, Hospital Universitario La Paz, 28046 Madrid, Spain; 12 Obstetrics and Gynecology Department, Santa Creu i Sant Pau University Hospital, 08025 Barcelona, Spain; 13 Facultat de Medicina, Universitat Autònoma de Barcelona, 08193 Barcelona, Spain

**Keywords:** congenital heart defects, fetal echocardiography, B-type natriuretic peptide, transforming growth factor beta, aortic stenosis, aortic coarctation, hypoplastic left heart syndrome, angiogenic factors, fetal cardiac remodeling

## Abstract

Fetal echocardiography has limited prognostic ability in the evaluation of left-sided congenital heart defects (left heart defects). Cord blood cardiovascular biomarkers could improve the prognostic evaluation of left heart defects. A multicenter prospective cohort (2013–2019) including fetuses with left heart defects (aortic coarctation, aortic stenosis, hypoplastic left heart, and multilevel obstruction (complex left heart defects) subdivided according to their outcome (favorable vs. poor), and control fetuses were evaluated in the third trimester of pregnancy at three referral centers in Spain. Poor outcome was defined as univentricular palliation, heart transplant, or death. Cord blood concentrations of N-terminal precursor of B-type natriuretic peptide, Troponin I, transforming growth factor β, placental growth factor, and soluble fms-like tyrosine kinase-1 were determined. A total of 45 fetuses with left heart defects (29 favorable and 16 poor outcomes) and 35 normal fetuses were included, with a median follow-up of 3.1 years (interquartile range 1.4–3.9). Left heart defects with favorable outcome showed markedly increased cord blood transforming growth factor β (normal heart median 15.5 ng/mL (6.8–21.4) vs. favorable outcome 51.7 ng/mL (13.8–73.9) vs. poor outcome 25.1 ng/mL (6.9–39.0), *p* = 0.001) and decreased placental growth factor concentrations (normal heart 17.9 pg/mL (13.8–23.9) vs. favorable outcome 12.8 pg/mL (11.7–13.6) vs. poor outcome 11.0 pg/mL (8.8–15.4), *p* < 0.001). Poor outcome left heart defects had higher N-terminal precursor of B-type natriuretic peptide (normal heart 508.0 pg/mL (287.5–776.3) vs. favorable outcome 617.0 pg/mL (389.8–1087.8) vs. poor outcome 1450.0 pg/mL (919.0–1645.0), *p* = 0.001) and drastically reduced soluble fms-like tyrosine kinase-1 concentrations (normal heart 1929.7 pg/mL (1364.3–2715.8) vs. favorable outcome (1848.3 pg/mL (646.9–2313.6) vs. poor outcome 259.0 pg/mL (182.0–606.0), *p* < 0.001). Results showed that fetuses with left heart defects present a distinct cord blood biomarker profile according to their outcome.

## 1. Introduction

Left-sided congenital heart defects (left-CHD) are the most common group of severe congenital heart defects and a major contributor to neonatal morbi-mortality [1]. In specialized settings, prenatal diagnosis is feasible in up to 90% of CHD [2]. Thus, fetal cardiology is now focused on predicting medium- and long-term cardiovascular outcomes. However, fetal echocardiography has shown limited prognostic ability, and therefore, new parameters are necessary to improve the prognostic evaluation of these anomalies in fetal life.

Several existing biomarkers are promising candidates. B-type natriuretic peptide (BNP) and its N-terminal precursor (NT-proBNP) have demonstrated utility in screening for CHD in newborns [3] and in predicting surgical outcomes in children with CHD [4]. Increased levels of Troponin, a specific marker of myocardial damage [5], have been found in the cord blood of neonates with single ventricle CHD and seem to relate to poorer prognosis [6]. Transforming growth factor beta (TGFβ), a cytokine implicated in different physiological and pathological responses [7,8], is expressed mainly by endothelial cells (at the cardiovascular level) and has been associated with fibrotic and hypertrophic remodeling (in heart failure and myocardial infarction), and extracellular matrix modulation in cardiac pressure overload [9,10,11]. However, it has never been studied in fetuses with CHD.

Additional biomarkers with a possible role in CHD are placental growth factor (PlGF) and its soluble receptor soluble fms-like tyrosine kinase-1 (sFlt1). PlGF regulates trophoblastic endothelial growth and is expressed by cardiomyocytes affecting ventricular remodeling in response to stress [12,13,14]. sFlt1 prevents the interaction of PlGF with cell receptors. An imbalance between PlGF and sFlt1 has been implicated in the pathogenesis of preeclampsia and intrauterine growth restriction [15]. Increased sFlt1 has also been detected in the cord blood of fetuses with CHD [16]. However, the role of PlGF and sFlt1 in CHD pathogenesis, associated cardiovascular remodeling, and/or prognosis has been insufficiently studied.

Thus, a differential expression profile of BNP, TGFβ, Troponin I, PIGF, and sFlt1 in cord blood could improve our understanding of cardiac remodeling in CDH and eventually help to identify those cases with poorer prognosis. We designed a prospective, multicenter study to measure cord blood biomarkers in fetuses with left-CHD and evaluate its association with disease severity.

## 2. Materials and Methods

### 2.1. Study Population

A prospective multicenter cohort study was conducted between 2013 and November 2019, including fetuses prenatally diagnosed with left-CHD. The cardiac outcome of these fetuses was regularly evaluated, and analysis was performed in terms of cardiac outcome. Fetuses with structurally normal hearts were also included as a non-exposed cohort. Pregnancies of women older than 18 years with accurate gestational age calculated by first-trimester crown–rump length [17] were considered eligible. Fetal standard ultrasound and echocardiography were performed in the third trimester, cord blood was obtained at delivery, and perinatal results and cardiac outcome data were collected.

Fetuses with left-CHD were recruited at the Fetal Cardiology Units from three tertiary Spanish referral centers: BCNatal (Hospitals Clinic and Sant Joan de Déu) and Hospital Vall d’Hebrón in Barcelona, and Hospital La Paz in Madrid. The group of left-CHD included aortic coarctation (CoA), aortic stenosis (AoS), hypoplastic left heart syndrome (HLHS), and complex left-CHD with multilevel left ventricular (LV) tract obstruction, including complete or incomplete Shone syndrome. Fetuses with CoA were included based on high prenatal echocardiographic suspicion (significant right dominance and aortic isthmus diameter < −2 z-score) [18] with postnatal diagnostic confirmation. AoS was defined based on fetal echocardiographic visualization of thickened aortic valve with abnormal Doppler flow and diastolic length of the left ventricle > −2 z-score at the time of evaluation [19]. HLHS was defined by an absent or small left ventricle (LV length < −2 or −3 z-score) with weak contractility with the absence of antegrade flow through the aortic valve and ascending aorta hypoplasia of variable degree [19]. This group included cases of mitral and/or aortic atresia and evolving HLHS secondary to severe aortic stenosis. Diagnosis of complex left-CHD was considered in fetuses with multilevel LV tract obstruction, including complete Shone syndrome (supravalvular mitral membrane, parachute mitral valve, muscular or membranous subvalvular aortic stenosis, and coarctation of aorta) and partial forms involving only two or three out of the four anomalies (incomplete Shone syndrome) [20]. Exclusion criteria in the left-CHD population included pre or postnatal diagnosis of additional major cardiac malformations, presence of major extracardiac malformations, or genetic anomalies. Minor cardiovascular anomalies (such as small ventricular septal defects, persistent left superior vena cava, or aberrant right subclavian artery) were not exclusion criteria. Clinical outcome was obtained from medical records at least one year after birth and reevaluated yearly if necessary. Prior to analysis, left-CHD cases were classified into favorable vs. poor cardiac outcome. Poor cardiac outcome was defined by the presence of at least one of the following: postnatal univentricular surgical palliation, need for heart transplant, or death related to CHD or its complications.

Control fetuses with structurally normal hearts were recruited from singleton uncomplicated and spontaneously conceived pregnancies attended at the Maternal-Fetal Medicine Department at BCNatal in Barcelona. They were matched for gestational age (±2 weeks) at scan with left-CHD fetuses. Exclusion criteria in this group included pre or postnatal diagnosis of CHD, extracardiac malformations, genetic anomalies or conditions potentially affecting fetal cardiac function, or cord biomarkers such as intrauterine growth restriction [21,22,23,24], maternal diabetes [25,26], exposure to toxins [27], or macrosomia [26].

### 2.2. Fetal Standard Ultrasound and Echocardiography

Standard obstetric ultrasound and fetal echocardiography were performed using a Siemens Sonoline Antares (Siemens Medical Systems, Malvern, PA, USA) or Voluson E8 (General Electric, Zipf, Austria) using a curved-array 2–6 MHz transducer. 

Fetal ultrasound was performed according to recommended guidelines and included calculation of estimated fetal weight [28] and centile [29], extracardiac and cardiac detailed examinations [30], and measurement of mean uterine artery, umbilical artery, and middle cerebral artery pulsatility indices [31]. The cerebroplacental ratio was calculated as the quotient between the median cerebral artery and umbilical artery pulsatility indices [32]. IUGR was defined as estimated fetal weight below the 3rd centile or below the 10th centile with abnormal maternal or fetal Doppler values [33]. 

The fetal cardiac morphometric assessment included cardiothoracic ratio [34,35], cardiac sphericity [35], atrial and ventricular-to-heart ratios [35], right-to-left ventricular ratio [35], and septal myocardium thickness [36,37]. Cardiac measurements were obtained in a two-dimensional apical or basal four-chamber view at end-diastole, except atrial areas, which were measured at end-systole. 

Cardiac function evaluation included heart rate, right ventricular fractional area change [38], LV shortening fraction [39], and filling and ejection time fractions [40]. LV filling and ejection time fractions were only measured when LV inflow or outflow waves could be identified, thus excluding cases with mitral and aortic atresia.

### 2.3. Cord Blood Biomarkers

Cord blood samples were obtained from the umbilical vein after cord clamping at birth. Plasma was separated from ethylenediaminetetraacetic acid-treated blood using centrifugation at 1400× *g* for 10 min at 4 °C. Serum was separated using centrifugation at 2000× *g* for 10 min at room temperature. Sample aliquots were immediately stored at −80 °C until assayed.

N-terminal precursor of B-type natriuretic peptide (NT-proBNP) and Troponin I concentrations was measured in plasma by electrochemiluminescence immunoassay using Siemens Atellica IM NT-proBNP and High Sensitivity Troponin I, respectively (Siemens Healthcare, Erlangen, Germany). Transforming growth factor β1 (TGFβ) was measured in serum by conventional ELISA assay Quantikine Human TGF-beta1 (R&D Systems, Minneapolis, MN, USA). Concentrations of placental growth factor (PlGF) and soluble fms-like tyrosine kinase-1 (sFlt1) were determined in serum by the fully automated Elecsys assays for sFlt-1 and PlGF on an electrochemiluminescence immunoassay platform Cobas analyzer (Roche Diagnostics, Mannheim, Germany). Concentrations of cord NT-proBNP, TGFβ, PlGF, and sFlt1 are presented as continuous variables (NT-proBNP, PlGF, and sFtl1 in pg/mL and TGFβ in ng/mL). Troponin I was treated as a dichotomous variable, and concentrations above the technical detection limit (>0.017 ng/mL) were considered positive [41].

### 2.4. Statistical Analysis

Data were analyzed using the IBM SPSS Statistics version 23 statistical package (IBM Corp., Armonk, NY, USA). The Shapiro–Wilk test of normality was performed for continuous variables. Continuous variables are presented as median (interquartile range) or mean ± standard deviation, as appropriate. For normally distributed variables, overall differences between study groups were examined using parametric analysis of variance (one-way ANOVA) followed by post hoc Bonferroni tests for pairwise comparison. Differences between study groups in non-normally distributed variables were analyzed using Kruskal–Wallis one-way ANOVA followed by post-hoc pairwise comparisons using the Dunn–Bonferroni approach. Categorical variables are presented as n (percentage %), and differences between the groups were compared using the appropriate chi-square test. When necessary and appropriate, Student’s *t*-test or Mann–Whitney U-test was also used. A significance level of 0.05 was used throughout.

Analysis of baseline variables for possible confounders (included maternal age, maternal body mass index, nulliparity, smoking, pregestational diabetes, gestational diabetes, preeclampsia, gestational age at delivery, birth weight, gender, mode of delivery, and echocardiographic diagnosis), identified an association between cesarean section and increased levels of NT-proBNP (cesarean section median 547.0 pg/mL (interquartile range 304.0–996.0) vs. vaginal delivery 893.0 pg/mL (488.5–1523.8), *p* = 0.026); thus, NT-proBNP was analyzed separately according to delivery route. The remaining cord biomarkers were not affected by the delivery route, and no other confounders were identified.

Logistic regression analysis adjusted by cardiac anatomy and delivery mode was performed, and receiver–operating characteristics (ROC) curves were constructed for cord blood biomarkers in the form of areas under the curve to investigate the presence of a cutoff value that might be predictive of outcome in those fetuses with expected biventricular outcome.

## 3. Results

### 3.1. Study Populations

**Figure 1:**
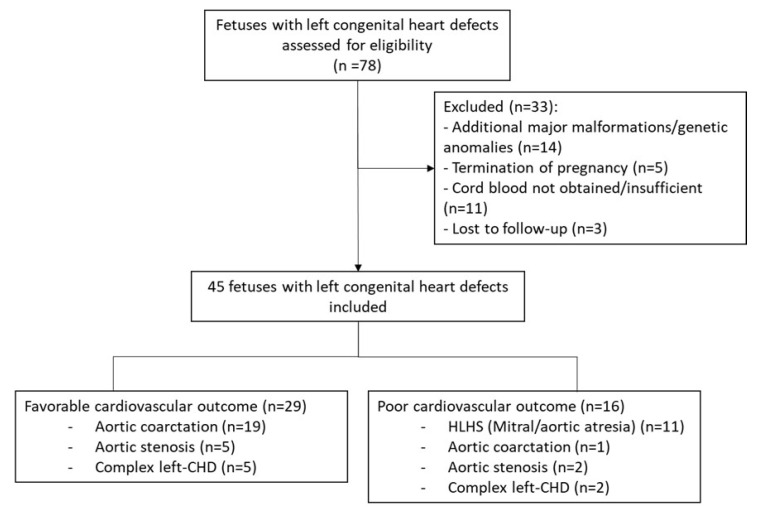
Flowchart of fetuses with left-side congenital heart defects included in the study.

The study population consisted of 35 healthy control pregnancies and 45 fetuses with left-CHD divided into favorable (*n* = 29) and poor (*n* = 16) cardiac outcome (Figure 1). Left-CHD with favorable cardiac outcome included 19 fetuses with CoA, 5 with AoS, and 5 cases of complex left-CHD. The poor cardiac outcome group was formed by 1 fetus with CoA, 2 with AoS, 2 cases of complex left-CHD, and 11 fetuses with HLHS. Univentricular surgical palliation was performed in the 14 newborns in the poor cardiac outcome group (87.5%), and there were 5 postnatal deaths (31.3%), of which 4 corresponded to surgical complications of the univentricular palliation procedure. The remaining case corresponded to a 5-month-old infant with complex left-CHD that died from severe pulmonary hypertension after biventricular management. One 3-year-old infant with complex left-CHD and biventricular management is awaiting a heart transplant at the time of writing. The medium follow-up of left-CHD cases was 3.1 years (interquartile range 1.4–3.9).

Maternal baseline characteristics, standard obstetrical ultrasound results, and perinatal results are shown in Table 1. The three study populations were similar in terms of maternal characteristics, gestational age at scan, estimated fetal weight, and centile and fetal Doppler. The mean uterine artery pulsatility index was more frequently abnormal (>95th centile) in the favorable cardiac outcome left-CHD group as compared to the normal heart group. Gestational age at delivery was similar across groups, and only two fetuses were born prematurely (one control and one favorable cardiac outcome left-CHD). Cesarean section was more frequent in the poor cardiac outcome group. Prevalence of pregnancy complications was similar in the three groups, including gestational diabetes, preeclampsia, birthweight < 3rd centile, and macrosomia. Birthweight, Apgar score, and umbilical artery pH were also similar. 

Cardiac morphological and functional evaluation in control, left-CHD with favorable and poor cardiac outcome fetuses are shown in Table 2. The cardiothoracic ratio was higher in both left-CHD groups compared to the normal heart group. Left atrial-to-heart and left ventricular-to-heart ratios were significantly lower in left-CHD, more markedly so in the poor cardiac outcome group. Concerning the right side of the heart, right atrial-to-heart and right ventricular-to-heart ratios were significantly increased in the poor cardiac outcome left-CHD compared to the normal heart group but not in the favorable cardiac outcome group. Right-to-left ventricular ratio (basal transverse diameter) was significantly increased in both groups of left-CHD. Septal thickness tended to be higher in left-CHD with poor prognosis; however, in post hoc pairwise comparison, the difference was not statistically significant (*p* = 0.068). Heart rate was similar in the three groups. The left shortening fraction was increased in the favorable cardiac outcome group of left-CHD and decreased in the poor cardiac outcome left-CHD compared to the normal heart group. The remaining functional parameters evaluated did not show significant differences between the three study populations. 

### 3.2. Cord Blood Biomarkers Results

**Figure 2:**
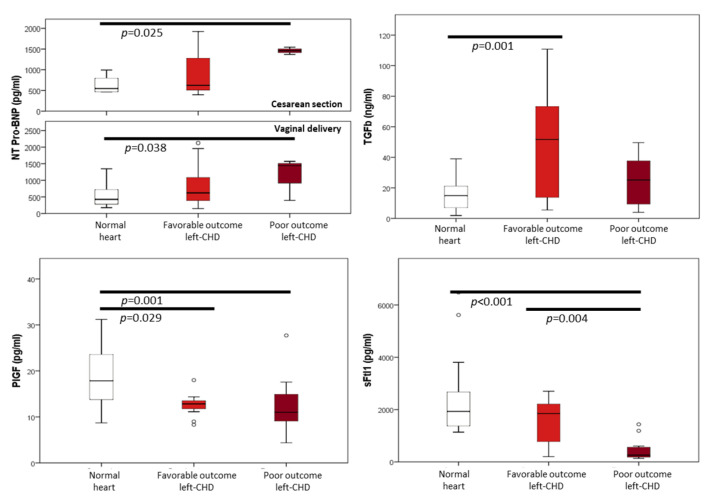
Concentrations of N-terminal precursor of B-type natriuretic peptide (NT-Pro-BNP), Troponin I, transforming growth factor β (TGFβ), placental growth factor (PlGF), and soluble fms-like tyrosine kinase-1 (sFlt1) in the cord blood of fetuses with normal heart and with favorable and poor outcome left congenital heart defects. Poor cardiac outcome is defined as univentricular circulation, heart transplant, or death. *p*-values are shown for significantly different (*p* < 0.05) comparisons.

Results of cord blood biomarkers in left-CHD and normal heart groups are presented in Table 3 and Figure 2. Compared to the normal heart group, left-CHD cases with favorable cardiac outcome presented preserved NT-proBNP concentrations. Cord blood Troponin I seemed to be more frequently elevated in the favorable cardiac outcome group, but this was not statistically significant. Fetuses with a favorable outcome also presented a markedly increased cord blood concentration of TGFβ and lower PlGF with no significant changes in sFlt1. Conversely, poor outcome left-CHD cases presented increased concentrations of NT-proBNP, which was independent of delivery mode. Troponin I showed positive results in a similar proportion to the control group. In the poor prognosis group, TGFβ concentrations were not significantly higher than in the normal heart group. Finally, PlGF was slightly reduced, while cord blood sFlt1 concentrations were drastically reduced in this group. These findings were independent of the specific anatomy.

**Figure 3:**
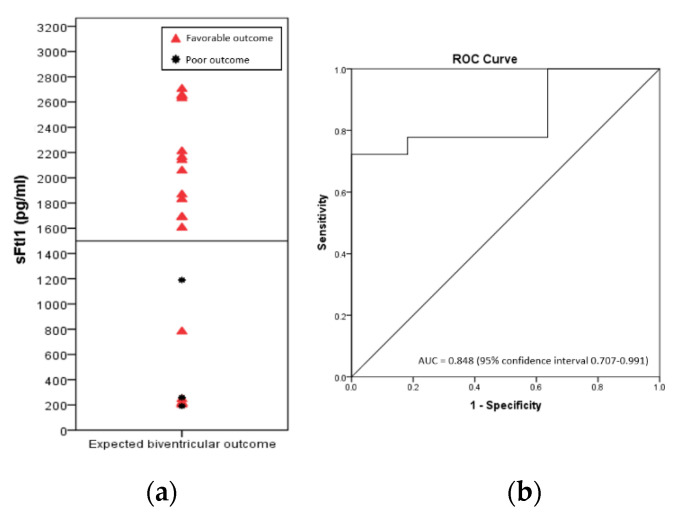
(**a**) Concentrations of soluble fms-like tyrosine kinase-1 (sFlt1) in the cord blood of control fetuses and fetuses with expected biventricular repair, classified according to outcome (red triangle, favorable outcome; black star, poor outcome. Reference line set at 1520.7 pg/mL identified at (**b**) ROC curve for sFlt1 as a predictor of favorable outcome in fetuses with expected biventricular outcome.

Logistic regression analysis and evaluation of ROC curves of concentration of cord blood biomarkers for the prediction of outcome in fetuses with expected biventricular repair, adjusted for cardiac anatomy and cesarean section, found that only sFlt1 was useful as an outcome predictor. The sFlt1ROC curve had an AUC of 0.848 (95% confidence interval 0.706–0.991) with a cutoff value of 1520.7, having a specificity of 100% and a sensitivity of 72% for a favorable outcome (Figure 3). 

## 4. Discussion

Cord blood cardiovascular biomarkers are differentially expressed according to the clinical outcome of left-CHD. Fetuses with left-CHD and favorable outcome showed an active cardiac remodeling biomarker profile, with an increase in TGFβ and Troponin I cord blood concentrations. Conversely, fetuses with left-CHD and poor outcome presented a compensatory and proangiogenic biomarker profile with increased NT-proBNP and decreased sFlt1 concentrations.

Prior studies reported increased NT-proBNP concentrations in children with systemic right ventricle and in single ventricle CHD before completion of staged palliation [42,43,44]. A different study evaluated cord blood NT-proBNP and Troponin T plus an echocardiographic score (including aortic size and cardiomegaly) to predict mortality in a variety of single ventricle CHD (approximately half were left-CHD) [6]. Both NT-proBNP and Troponin T were found to be useful predictors in this setting [6]. While obvious differences exist between both studies [6] in terms of CHD included and outcome definition, increased NT-proBNP seems to be reflecting fetal adaptation to ventricular stretching or hypoxia and correlate with the disease severity and outcome. 

Positive cord blood Troponin I was more frequent in left-CHD with favorable cardiac outcome than in fetuses with normal hearts or fetuses with poor outcome. Differences were not statistically significant, and these results must be interpreted with caution as the normal heart group presented higher positive rates of Troponin I than previously reported in healthy fetuses [41,45,46]. In a prior study, Troponin I was detected more frequently in children with pressure overload CHD (CoA, AoS, and pulmonary stenosis) rather than volume overload CHD (atrial septal defect and patent ductus arteriosus) [45]. In the present study, most cases of pressure overload (AoS and complex left-CHD) presented a favorable outcome, while the poor outcome group included mostly fetuses with HLHS (right ventricular volume overload), which may explain our findings. Increased levels of Troponin I in cord blood might reflect endocardial hypoxia and myocardial damage secondary to increased end-diastolic ventricular pressure and wall stress [47]. 

To our knowledge, this is the first study to evaluate cord blood concentrations of TGFβ in fetuses with CHD, showing increased TGFβ concentrations in left-CHD with favorable outcome. High levels of circulating TGBβ are usually associated with increased endothelial shear stress, secondary to abnormal fluid dynamics within blood vessels [48,49]. All cases in the favorable outcome group presented anatomical impediments to normal blood flow and, thus, turbulent flow and increased endothelial shear stress that could stimulate the production of TGFβ by endothelial cells. In addition, increased TGFβ expression has been related to extracellular matrix deposition and fibrosis and arterial intima-media thickening and can be detected by immunohistochemistry at the coarctation site, especially in infants with a closed arterial duct [9,10,50]. On the other hand, TGFβ concentrations were not increased in the poor outcome group, which was mostly composed of fetuses with full cardiac output handled by the right heart and laminar flow through the pulmonary artery.

Cord blood concentrations of PlGF were found to be modestly decreased in left-CHD compared to normal heart fetuses. However, sFlt1 was markedly decreased in the poor outcome group conferring a proangiogenic profile of PlGF and sFlt1. Such low levels of sFlt1 had not been described previously in pregnancy, and their cause is unclear. However, sFlt1 is down-regulated by hypoxia [51], and therefore, our findings might be explained by the hypoxic damage of the left ventricular endocardium of fetuses with poor outcome left-CHD [52,53]. A previous study including a mixed group of CHDs, including atrioventricular septal defects (A-V defects), conotruncal anomalies, and left-CHD reported similar PlGF but increased sFlt1 cord blood levels in CHD [16]. This inconsistency might be explained by the different types of CHD included in both studies. The presence of a high proportion of A-V defects and conotruncal anomalies, as well as missing information in terms of left-CHD types and/or severity, precludes direct comparison between both studies. 

Finally, cord blood sFlt1 was identified for the first time as a possible outcome predictor in fetuses with expected biventricular repair and may eventually help to identify cases at risk of an unexpectedly poor prognosis despite favorable anatomy. Naturally, due to the limited number of cases in this subgroup, confirmation in larger cohorts is required prior to clinical application. A larger number of cases might also demonstrate the utility of NT-proBNP or TGFβ in these patients in which a poor prognosis comes as a surprise.

### Strengths and Limitations

To the best of our knowledge, this is the first study to evaluate cord blood biomarkers in a specific set of CHDs and to evaluate TGFβ in the cord blood of fetuses with CHDs. It is also the first study to identify TGFβ and specially sFlt1 as potentially useful cardiovascular biomarkers in left-CHD.

This is mainly an exploratory study, and therefore, the eventual clinical application of our findings will likely be distant. The main limitation of this (and other studies) is the limited number of cases that precludes the evaluation of these biomarkers in each specific type of CHD. Interpretation of results is also limited by the presence of mixed pathologies in the favorable and poor outcome left-CHD groups. Cord blood biomarker levels were analyzed after adjustment for possible confounders; however, additional confounders might exist. Additionally, levels of Troponin I in the cord blood of normal fetuses were higher than previously reported [41,45,46], which limits the interpretation of our results.

## 5. Conclusions

This study provides evidence of a differential biomarker profile in left-CHD according to its outcome. It opens new opportunities for improving prognosis prediction and potentially helping in prenatal monitoring, neonatal management, and parental counseling. If confirmed in future studies, these biomarkers could potentially be useful as a prognosis tool in prenatal or neonatal life. In addition, future studies are warranted to study cardiovascular biomarkers in different matrices such as amniotic fluid or maternal blood. Long-term multicenter studies might be able to include enough patients and allow specific evaluation and analysis of each individual type of left-CHD. Additionally, the investigation of these biomarkers in the cord blood of other CHDs might also yield interesting results.

## Figures and Tables

**Table 1:** Maternal baseline characteristics, standard ultrasound, and perinatal results in the study populations.

	Normal Heart	Favorable Outcome Left-CHD (*n* = 29)	Poor Outcome Left-CHD (*n* = 16)	*p*
	(*n* = 35)			
**Maternal baseline characteristics**				
**Maternal age (years)**	33.2 ± 5.2	31.9 ± 6.7	33.7 ± 5.6	0.567
**Maternal body mass index**	23.2 ± 3.2	25.6 ± 3.7	25.6 ± 5.4	0.095
**Nulliparity**	17 (48.6%)	15 (51.7%)	7 (43.8%)	0.877
**Smoking**	4 (11.4%)	4 (13.8%)	1 (6.3%)	0.745
**Pregestational diabetes**	0 (0%)	0 (0%)	0 (0%)	1.000
**Standard Obstetrical Ultrasound**				
**Gestational age at scan (weeks)**	33.1 (29.6–35.1)	33.1 (32.3–34.3)	33.1 (32.3–34.3)	0.646
**Female fetus**	14 (40%)	15 (51.7%)	6 (37.6%)	0.548
**Estimated fetal weight (g)**	2011 ± 681	2037 ± 560	1918 ± 680	0.933
**Estimated fetal weight centile**	58.0 (27.0–76.0)	32.5 (18.3–73.0)	84.0 (43.0–86.5)	0.132
**Intrauterine growth restriction**	0 (0%)	2 (6.9%)	0 (0%)	0.165
**Umbilical artery PI**	1.0 (0.9–1.1)	1.0 (0.8–1.3)	1.0 (0.8–1.3)	0.723
**Umbilical artery PI >95th centile**	0 (0%)	1 (3.4%)	0 (0%)	0.410
**Median cerebral artery PI**	2.0 ± 0.4	1.8 ± 0.3	1.7 ± 0.1	0.087
**Median cerebral artery <5th centile**	1 (2.9%)	1 (3.4%)	0 (0%)	0.765
**Cerebro-placental ratio**	2.0 ± 0.5	1.8 ± 0.4	1.8 ± 0.5	0.111
**Cerebro-placental ratio <5th centile**	2 (5.7%)	2 (6.7%)	0 (0%)	0.577
**Mean Uterine Artery PI**	0.7 (0.5–0.8)	0.9 (0.7–1.1)	0.7 (0.7–0.9)	0.074
**Mean Uterine Artery PI >95th centile**	1 (2.9%)	5 (17.2%)	0 (0%)	0.042
**Perinatal Results**				
**Gestational age delivery (weeks)**	39.8 (38.6–40.5)	39.7 (38.4–40.3)	40.0 (39.5–40.0)	0.723
**Cesarean section**	8 (22.8%)	6 (21.4%)	7 (63.6%) *	0.019
**Spontaneous preterm birth < 37 weeks**	1 (2.9%)	1 (3.4%)	0 (0%)	0.765
**Gestational diabetes**	3 (8.6%)	3 (10.3%)	2 (12.5%)	0.907
**Preeclampsia**	0 (0%)	1 (3.4%)	1 (6.3%)	0.381
**Birth weight (g)**	3256 ± 416	3173 ± 525	3275 ± 415	0.660
**Birth weight < 3rd centile**	0 (0%)	2 (6.9%)	0 (0%)	0.165
**Birth weight > 4000 g**	0 (0%)	1 (3.4%)	0 (0%)	0.410
**5-min Apgar score < 7**	0 (0%)	1 (3.4%)	0 (0%)	0.410
**Umbilical artery pH**	7.22 (7.17–7.27)	7.25 (7.18–7.27)	7.26 (7.20–7.30)	0.416
**Cardiovascular outcome**				
**Follow-up (years)**	-------	3.1 (1.4–3.9)		-------
**Univentricular circulation**	-------	-------	11 (68.8%)	-------
**Cardiac transplant**	-------	-------	1 (6.3%)	-------
**Death**	-------	-------	5 (31.3%)	-------

Data are presented as mean ± standard deviation, median (interquartile range), or n (percentage %) as appropriate. Left-CHD: left-sided congenital heart disease. Poor outcome defined as postnatal univentricular surgical palliation, need for heart transplant, or death. PI: pulsatility index. Intrauterine growth restriction is defined as estimated fetal weight below the 3rd centile or below the 10th centile with abnormal maternal or fetal Doppler values. *p* is shown for the ANOVA between the 3 groups. * *p* < 0.05 in pairwise comparisons in ANOVA post hoc Bonferroni or Dunn–Bonferroni tests of favorable and poor outcome vs. normal heart.

**Table 2:** Fetal echocardiographic results in the study populations.

Parameter	Normal Heart	Favorable Outcome	Poor Outcome	*p*
		Left-CHD	Left-CHD	
	(*n* = 35)	(*n* = 29)	(*n* = 16)	
**Cardiac morphometric parameters**				
**Gestational age at scan (weeks)**	33.1 (29.6–35.1)	33.1 (32.3–34.3)	33.1 (32.3–34.3)	0.646
**Cardio-thoracic ratio**	0.28 ± 0.04	0.30 ± 0.05 *	0.32 ± 0.05 *	0.002
**Cardiac sphericity index**	1.2 (1.1–1.3)	1.2 (1.1–1.3)	1.1 (1.0–1.3)	0.087
**Left atrial-to-heart ratio**	0.15 ± 0.04	0.13 ± 0.03 *	0.10 ± 0.03 *	<0.001
**Left ventricular-to-heart ratio †**	0.23 (0.19–0.28)	0.15 (0.14–0.20) *	0.11 (0.03–0.14) *	<0.001
**Right atria-to-heart ratio**	0.17 ± 0.05	0.20 ± 0.03	0.23 ± 0.08 *	0.001
**Right ventricle-to-heart ratio**	0.23 (0.20–0.27)	0.24 (0.21–0.26)	0.29 (0.25–0.36) *	0.016
**Right-to-left ventricular ratio (basal)**	1.0 (0.9–1.1)	1.4 (1.3–1.7) *	2.1 (1.9–3.7) *	<0.001
**Septal wall thickness (mm)**	3.1 (2.6–3.7)	3.5 (3.0–4.1)	3.6 (3.4–3.9)	0.040
**Cardiac function parameters**				
**Heart rate**	141 (134–144)	137 (131–145)	140 (131–141)	0.693
**Left ventricular Shortening Fraction ‡**	36.7 ± 8.8	44.3 ± 12.9 *	21.0 ± 11.2 *	<0.001
**Left filling time fraction ‡**	43.2 ± 4.9	42.0 ± 3.6	41.2 ± 14.6	0.633
**Left ejection time fraction ‡**	41.0 (338.9–43.4)	42.1 (37.9–44.8)	45.1 (38.4–64.6)	0.282
**Right ventricular Fractional Area Change**	30.4 ± 8.4	30.7 ± 11.2	35.6 ± 10.6	0.371
**Right filling time fraction**	40.4 (37.6–43.9)	42.3 (38.4–44.2)	38.9 (35.4–43.4)	0.480
**Right ejection time fraction**	42.6 ± 3.3	41.8 ± 3.8	43.0 ± 4.1	0.577

Data are presented as mean ± standard deviation or median (interquartile range). Left-CHD: left-sided congenital heart disease. Poor cardiac outcome defined by the presence of postnatal univentricular circulation, need for heart transplant, or death. † Measured when the left ventricle was visible (*n* = 10 in the poor outcome group). ‡ Measured when left ventricular inflow/outflow was detectable (*n* = 5 in the poor outcome group). P is shown for the ANOVA between the 3 groups. * *p* < 0.05 in pairwise comparisons in ANOVA post hoc Bonferroni or Dunn–Bonferroni tests of favorable and poor outcome vs. normal heart.

**Table 3:** Concentrations of N-terminal precursor of B-type natriuretic peptide (NT-Pro-BNP), Troponin I, transforming growth factor β (TGFβ), placental growth factor (PlGF), and soluble fms-like tyrosine kinase-1 (sFlt1) in the cord blood of control fetuses and fetuses with favorable versus poor outcome left congenital heart defects.

Cord Blood Cardiovascular Biomarkers	Normal Heart	Favorable Outcome	Poor Outcome	*p*
		Left-CHD	Left-CHD	
	(*n* = 35)	(*n* = 29)	(*n* = 16)	
**NT Pro-BNP (pg/mL)**	508.0 (287.5–776.3)	617.0 (389.8–1087.8)	1450.0 (919.0–1645.0) *	0.001
**TroponinI (% positive)**	7/31 (22.6%)	9/19 (47.4%)	2/9 (22.2%)	0.153
**TGFβ (ng/mL)**	15.0 (6.8–21.4)	51.7 (13.8–73.9) *	25.1 (6.9–39.0)	0.001
**PlGF (pg/mL)**	17.9 (13.8–23.9)	12.8 (11.7–13.6) *	11.0 (8.8–15.4) *	<0.001
**sFlt1 (pg/mL)**	1929.7 (1364.3–2715.8)	1848.3 (646.9–2313.6)	259.0 (182.0–606.0) *	<0.001

Data are presented as median (interquartile range) or n (percentage). Left-CHD: left-sided congenital heart disease. Troponin I was considered positive when higher than 0.017 ng/mL. P is shown for the ANOVA or chi-square tests between the 3 groups. * *p* < 0.05 in pairwise comparisons in ANOVA post hoc Dunn–Bonferroni tests of favorable and poor outcome vs. normal heart.
